# Spatial-temporal analysis of urban-rural differences in the development of elderly care institutions in China

**DOI:** 10.3389/fpubh.2022.1086388

**Published:** 2022-12-22

**Authors:** Xiang Li, Chen Li, Yi Huang

**Affiliations:** ^1^School of Economics and Management, Sanming University, Sanming, China; ^2^School of Management, Shanghai University of Engineering Science, Shanghai, China; ^3^School of Geographic Sciences, Nantong University, Nantong, China

**Keywords:** elderly care institutions, urban-rural differences, spatial-temporal analysis, local spatial autocorrelation, China

## Abstract

**Background:**

Aging is both a sign of rising life expectancy per capita and social progress, and a challenge for society. Due to the decline in physiological functions, the rate of illness has increased significantly, leading to a rise in demand for healthcare, life care and other elderly care. With the overlapping impact of an aging population, advanced aging, empty nesting families and the weakening of traditional elderly care functions, the issue of elderly care for the empty nesters, the elderly alone and the disabled has become more prominent and has become a focal point of concern for all sectors of society. As an important supplement to the elderly care service system, institutional care, together with home care, community care and rural care, are mutually complementary.

**Methods:**

The study establishes a panel database of urban and rural elderly-care institutions in 276 cities from 2010 to 2016, and uses comprehensive measurements to reveal the spatial-temporal changes of urban and rural elderly care institutions in China.

**Results:**

First, in terms of spatial pattern, the overall score of elderly care institutions in urban areas shows a “double-high” spatial pattern of higher scores in coastal areas than inland areas, and higher scores in urban areas than in rural areas. In terms of the differences in the scores of secondary indicators, the eastern urban areas have higher scores than the rural areas for the indicators of facilities construction and nursing staff of elderly institutions, while the eastern rural areas have higher scores than their urban counterparts for the indicators of service recipients of elderly institutions. Second, in terms of temporal change, there is a clear “urban progress and rural regression” in the evolution of China's elderly care institutions. Third, in terms of spatial and temporal evolution, there is a clear spatial autocorrelation in the composite scores of urban and rural elderly care institutions in China, and the spatial autocorrelation of the composite scores of elderly care institutions shows a clustering pattern.

**Discussion:**

The contradiction between the limited ability to pay of the elderly people staying in elderly care institutions and the huge demand for elderly care services is bound to affect the sustainability of the development of public elderly care institutions in both urban and rural areas. Due to historical factors, the marketisation of elderly care institutions in China started late and the marketisation of elderly care is not high. As the population ages, China's elderly-care institutions have begun to transform from public institutions of a welfare nature to those with some market mechanisms, but the overall transformation has been slow, resulting in the service guarantee system of elderly-care institutions lagging far behind the actual needs of the elderly. The long-term development of elderly care institutions must introduce market mechanisms, enhance the endogenous dynamics of elderly care institutions, correctly handle the relationship between fairness and efficiency of elderly care services, and improve the professionalism, income and treatment of elderly care staff while compensating for the lack of development of elderly care institutions and the inadequate layout of space, so as to continuously improve the service quality of elderly care institutions.

## Introduction

The report World Population Prospects 2022, prepared by the United Nations Department of Economic and Social Affairs, projects that the global population will grow to about 8.5 billion by 2030, reach 9.7 billion in 2050, peak at about 10.4 billion in the 1980s and remain at that level until 2100, with continued increases in global average life expectancy, superimposed on declining fertility rates, exacerbating global population aging (1). China officially entered an aging population society in 2000. As we enter the 21st century, the number and size of China's elderly population is rapidly expanding. Data from the seventh population census shows that the number of elderly people aged 60 and above in China will reach 260 million in 2020, accounting for 18.70% of the total population (2). From now until the middle of the 21st century, it will be a period of rapid development of population aging in China. It is predicted that by 2050, China's elderly population will be nearly 380 million, accounting for about 1/3 of the total population (3). Due to the different living environment, retirement protection and family retirement resources of the elderly in urban and rural areas, it is important to grasp the law of development of urban and rural retirement institutions to actively cope with the aging population, healthy urban and rural retirement service systems and achieve a sense of security for the elderly.

Essentially, the challenges of population aging stem mainly from the contradictions arising from the incompatibility between the age structure of the aging population and the existing socio-economic system (4). As the physical functions of the elderly tend to naturally age and decline, coupled with the superimposed impact of advanced aging, empty nesting families and the weakening of traditional elderly care functions, it exacerbates the problem of elderly care for the empty nesters, the lost and the disabled (5), and to ensure a decent quality of life and life for the elderly in their old age, it is necessary to accelerate the establishment of a sound diversified, diverse and accurate elderly care service guarantee system (6). As an important supplement to the elderly care service system, institutional elderly care, together with home-based elderly care, community-based elderly care and rural elderly care, is an integral part of and complements each other (7). To build a coordinated elderly care service system of “home-based elderly care, community-based elderly care and institutional elderly care,” it is necessary to develop a “universal” elderly care service system. The development of an “inclusive” urban and rural elderly care service system, and the improvement of public elderly care institutions for the underprivileged and inclusive (8) are of great value to the quality of life and protection of the rights and interests of the elderly. According to the 14th Five-Year Plan for National Economic and Social Development of the People's Republic of China and the Outline of Vision 2035, “deepening the reform of public elderly institutions” is an important element in improving the elderly service system (9), the elderly institutions in our article refer to public elderly institutions, which are an important part of the elderly service system (10).

Academics have conducted a lot of research on elderly care institutions and their related services, focusing on the assessment of the current situation, influencing factors, matching supply and demand and countermeasures for elderly care services. In terms of current situation assessment, physical condition, daily activity patterns, spatial-temporal behavior, mental state, activity capacity and other physical and mental characteristics of the elderly are explored and assessed (11–16). In terms of influencing factors, the mechanisms of machine learning, social participation, cognitive ability, living conditions and community environment on the health of the elderly are analyzed (17–22). In terms of matching supply and demand, the demand for health care in old age is analyzed (23). In terms of countermeasure research, intervention strategies are provided from the perspectives of medical care integration, matching supply and demand, legislative optimization, and equalization of elderly care services, technological tools, Internet of Things devices, life intervention mechanisms, and medical diagnosis (24–30). In terms of spatial analysis, scholars have conducted analyses at different spatial scales, which are divided into four main categories: first, spatial-temporal analysis at the national scale. A national evaluation database of elderly care institutions at the sub-prefecture level was established to assess the density distribution and level of equalization of their elderly care services (31), reveal the urban-rural differences in the supply, demand and utilization of community-based elderly care services in the east, middle and west (32), explain the factors affecting the development of elderly care institutions in China (33) and predict the future changes of elderly people in need of care at different income levels (34). Second, spatial-temporal analysis at the regional scale. To reveal the spatial pattern of elderly care institutions in city clusters (35) and to explore the spatial scale spatial-temporal evolution characteristics of elderly care resource allocation in each province (36). Third, spatial-temporal analysis at the urban scale. The accessibility, supply and demand matching, spatial layout and optimal evaluation of urban aged-care institutions are evaluated (37–43). Fourth, community-scale spatial-temporal analysis. GIS technology is used to analyses the spatial distribution of elderly institutions at the community scale, to solve the accuracy of the spatial allocation of elderly institutions, and to provide countermeasure suggestions for community-based home care (44–47).

From the existing research results, most of the studies have conducted in-depth discussions on the spatial layout and accessibility of elderly care institutions, and most of the studies have analyzed the planning of elderly care facilities and the satisfaction of elderly people with elderly care facilities or the assessment of the health environment of the elderly, but relatively speaking, the analysis of the differences between urban and rural elderly care institutions is insufficient, especially the comparative analysis of the time-series data of urban and rural elderly care institutions is lacking. In view of this, this study analyzes the spatial layout of urban and rural aged-care institutions in China from 2010 to 2016 with the help of a GIS spatial analysis platform to reveal the characteristics of the spatial and temporal differences in the development of urban and rural aged-care institutions in China, and to provide ideas and suggestions for the rational spatial layout of urban and rural aged-care institutions, so as to promote the sustainable and healthy development of aged-care institutions.

## Research methods and indicator systems

### Object of study

**Figure 1:**
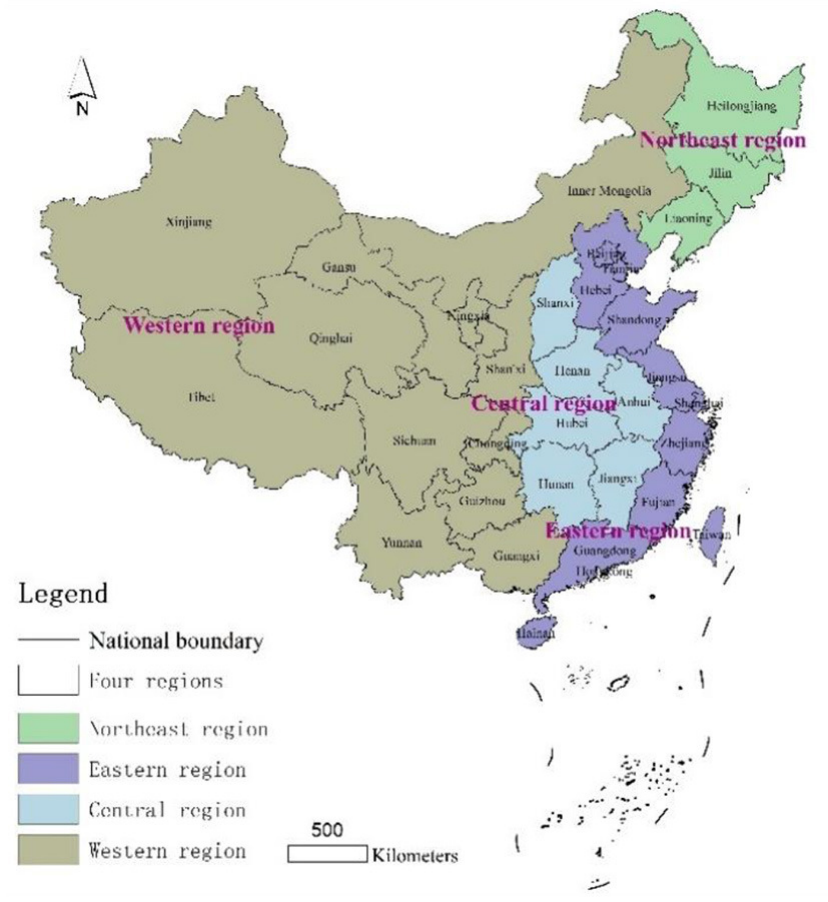
Schematic diagram of the study area.

The research object of this paper is the elderly institutions in urban areas and rural areas of China. The number of cities at prefecture level and above involved in the northeast, east, central and west regions are 33, 86, 77, and 80 respectively, which can reflect the development of most of the urban and rural elderly institutions in the region. The four regions are divided according to the outline of the Eleventh Five-Year Plan for National Economic and Social Development in 2006. “The Eleventh Five-Year Plan proposes to adhere to the overall regional development strategy of promoting the development of the western region, revitalizing old industrial bases such as the northeastern region, promoting the rise of the central region and encouraging the eastern region to take the lead in development, improve the mechanism of regional coordination and interaction, and form a reasonable regional development pattern” (48), which we have also discussed (49–52). China's eastern region includes 13 provinces, municipalities directly under the Central Government and special administrative regions, including Hebei, Beijing, Tianjin, Shandong, Jiangsu, Zhejiang, Shanghai, Guangdong, Hainan, Fujian, Taiwan, Hong Kong and Macao; the central region includes six provinces, including Shanxi, Henan, Anhui, Hubei, Jiangxi and Hunan; the western region includes Chongqing, Sichuan, Shaanxi, Yunnan, Guizhou, Guangxi, Gansu, Qinghai, Ningxia, Tibet, Xinjiang and Inner Mongolia and other 12 provinces, autonomous regions and municipalities directly under the central government, and the northeast region includes three provinces, including Heilongjiang, Liaoning and Jilin (Figure 1).

### Research design

**Figure 2:**
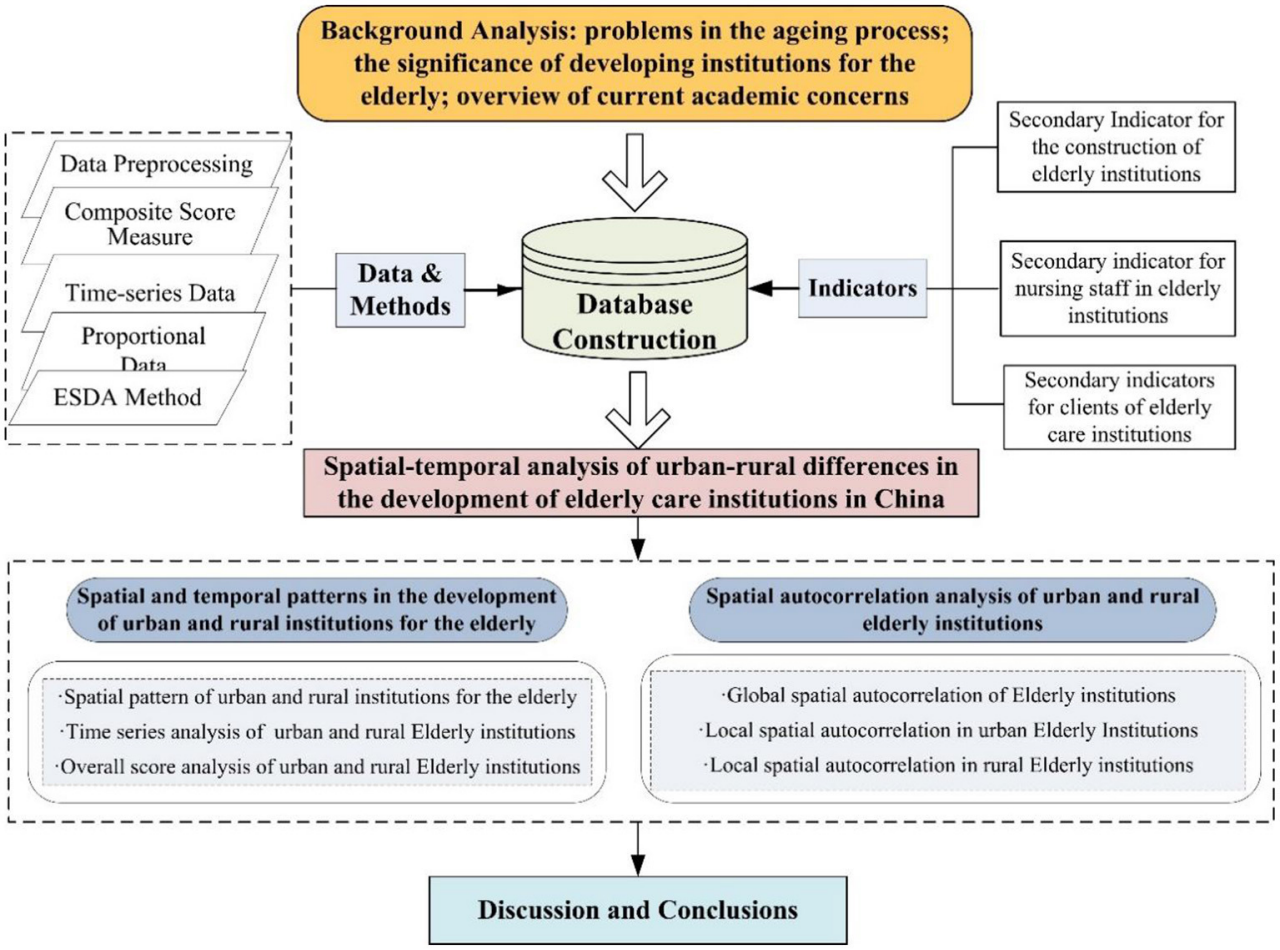
Technical route.

Based on the background analysis, this study uses panel data on urban and rural aged-care institutions to construct an evaluation index system in three aspects: aged-care institution construction, aged-care institution nursing staff and aged-care institution service recipients, to comprehensively measure the level and spatial and temporal characteristics of aged-care institution development in urban and rural China, to compare the differences between the level, scale and structure of urban and rural aged-care institution development, to analyze the local spatial autocorrelation differences between urban and rural aged-care institution development, and to discuss the findings and draw key conclusions (Figure 2).

### Data sources

The data on evaluation indicators for the development of elderly care institutions in urban and rural areas are all sourced from the China Civil Affairs Statistical Yearbook 2011–2017 compiled by the Ministry of Civil Affairs. Important indicators such as the floor area of elderly institutions only started to appear in the 2011 Civil Affairs Statistical Yearbook, and in terms of data availability, the 2018 and subsequent Civil Affairs Statistical Yearbooks became data on the spatial scale of the province, with urban and rural data by prefecture-level city not yet found. Therefore, the time scale of the study is 2010–2016. Although some of the indicators are very important, such as the level of professional qualifications, the classification of the nature of personnel, the number of volunteer hours and the number of volunteer visits in elderly institutions at the end of the year, the lack of statistics for continuous data from 2010–2016 forced the discarding of this part of the indicators. Some of the indicators, such as the number of rehabilitation and medical outpatient visits and the number of beds in honor rooms, are difficult to reflect the spatial and temporal trends in the pre-analysis process. In addition, during the data processing process, some of the indicators have been discarded. In addition, in the process of data processing, some cities are missing data, so we mainly use methods such as averaging and trend extrapolation to ensure the integrity and continuity of the data to the greatest extent possible.

### Indicator system

From the selection of indicators in the existing literature, most studies focus on the construction of the hardware environment and facilities of aged-care institutions, on the accessibility of aged-care facilities, and on the evaluation of indicators such as the number of beds in aged-care institutions and the area of aged-care institutions, but the attention to indicators of the software environment of aged-care institutions is relatively less abundant. In our research design, we take into account two major factors, namely the supply side of elderly institutions and the demand side of elderly institutions, and select three comprehensive measures of indicators at the levels of elderly institutions' facility construction, elderly institutions' nursing staff and elderly institutions' service recipients. The facility construction of elderly care institutions is the indicator of hardware facilities, while the nursing staff of elderly care institutions and service recipients of elderly care institutions are the indicators of soft environment. The nursing staff of elderly institutions are the service suppliers, while the service recipients of elderly institutions are the service demanders. The indicator system for the development of elderly-care institutions is constructed on three levels: the construction of facilities, the nursing staff of elderly-care institutions and the service users of elderly-care institutions, which reflect the construction of hardware facilities, the development of software conditions and the situation of elderly people living in elderly-care institutions respectively. The three-level indicators are refined to the two-level indicators, with a total of 17 three-level indicators, involving 6 quantitative indicators such as the number of elderly care institutions and 11 proportional indicators such as age structure, revealing the time-series characteristics of the development of urban and rural elderly care institutions from two dimensions: scale and structure.

In terms of setting the weight of the indicators, firstly, the weight of the 17 indicators was taken as the average value, i.e., the weight of the three-level indicator was 0.0588; secondly, considering the structural effect of the proportion indicators, the weights of the three second-level indicators of age structure, the classification of people living in elderly institutions by nature and the classification of people living in elderly institutions by self-care ability were summed up respectively, and the weights of the three-level indicators were redistributed. The sum of the weights of the above three secondary indicators is 0.1765, and the weights assigned to the age structure tertiary indicators of the proportion of persons elderly 35 and below, the proportion of persons elderly 36–55 and the proportion of persons elderly 56 and above are 0.5, 0.3 and 0.2 respectively. The weight given to the three-level indicators of the proportion of persons admitted to residential care institutions by nature, the proportion of persons with “three noes” and the proportion of self-financed persons, are 0.2, 0.3, and 0.5 respectively. The weights given to the three-level indicators of the proportion of people who are fully self-care, the proportion of people who are semi-self-care and the proportion of people who cannot take care of themselves according to their self-care ability are 0.2, 0.35, and 0.45 respectively. The above weights were multiplied by the weights of the corresponding secondary indicators to obtain the new weights of the tertiary indicators (Table 1).

**Table 1:** Indicator system for the development of elderly care institutions.

**Primary indicators**	**Secondary indicators**	**Tertiary indicators**	**weights**
Construction of facilities	Number of elderly care institutions	Number of elderly care institutions	0.0588
	GFA of elderly care facilities	GFA of elderly care facilities	0.0588
	Number of beds at end of year	Number of beds at end of year	0.0588
Nursing staff of elderly-care institutions	Number of employees at end of year	Number of employees at end of year	0.0588
	Gender structure	Proportion of female caregivers	0.0588
	Level of education	Percentage of university education	0.0588
	Age structure	Proportion of persons aged 35 and under	0.0882
		Proportion of persons aged 36-55 years	0.0529
		Proportion of persons aged 56 and over	0.0353
Service users of elderly-care institutions	Admission to elderly care institution	Average number of days in elderly care institution per year	0.0588
		Number of people in elderly care institution at the end of the year	0.0588
	Classification of people in elderly care institution by nature	Proportion of beneficiaries	0.0353
		Proportion of “three noughts”	0.0529
		Proportion of self-financing staff	0.0882
	Classification of people in elderly care institution according to their ability to care for themes	Proportion of fully self-care workers	0.0353
		Proportion of semi-self-care persons	0.0618
		Proportion of people who cannot care for themselves	0.0794

The Delphi method was used to construct the weights and expert opinion was consulted to give the weights and the reasons for re-weighting each of the nine proportional indicators. Firstly, young people elderly 35 and under are given relatively high weights if they can give priority to employment in elderly care institutions, representing the dynamism of the sector and reflecting the development momentum of elderly care institutions. Secondly, the proportion of self-funded staff is given a higher weighting, reflecting the trend of market-oriented reform of elderly care institutions. The government can play a bottom-up role in public elderly institutions, helping some of the “three have-nots” to solve their elderly care problems, but the development of elderly institutions can only have a lasting vitality if they are properly profitable. Thirdly, the proportion of semi-self-care workers and the proportion of those who cannot take care of themselves are given higher weight in view of the changes in family structure today. Children are under great pressure at work and are faced with support work such as looking after children and elderly people who are semi-self-care or unable to look after themselves, and the difficulty factor of balancing work and family care is high, which urgently requires the help of external environments such as elderly care institutions, and therefore indicators such as the proportion of semi-self-care persons staying in elderly care institutions and the proportion of persons unable to look after themselves are also assigned higher weights.

### Research methods

#### Composite score measure

##### Data pre-processing

Due to the differences between data units and data attributes in the indicator system, direct comparison between data with different attributes is not convenient, so the raw data need to be pre-processed. Considering the positive and negative attributes of the indicators, the raw data are dimensionless by using the method of extreme difference standardization (53).


(1) $$\{\begin{array}{l} d_{ij}=\frac{x_{ij}-x_{ij\min}}{x_{ij\max}-x_{ij\min}}(\text{PositiveIndicator})\\ d_{ij}=\frac{x_{ij\max}-x_{ij}}{x_{ij\max}-x_{ij\min}}(\text{NegativeIndicator}) \end{array}$$

Where, *d*_*ij*_ is the standardized value of indicator *ij* of the comprehensive evaluation index of the development of urban and rural elderly-care institutions. *x*_*ijmax*_ is the maximum value of indicator *ij* of the comprehensive evaluation index of the development of urban and rural elderly institutions, and *x*_*ijmin*_ is the minimum value of indicator *ij* of the comprehensive evaluation index of the development of urban and rural elderly institutions. *x*_*ij*_is the original value of the indicator. *d*_*ij*_ reflects the level of each indicator reaching the target, *d*_*ij*_ tends to 0 as the worst, *d*_*ij*_ tends to 1 as the best, and 0≤*d*_*ij*_≤1. The indicators in this study are all treated as positive attributes.

##### Composite score measurement


(2) $$\begin{array}{l} Z_{i}=\sum \omega_{i}{d_{i}}_{j} \end{array}$$

Where, Zi denotes the comprehensive score of urban and rural elderly institutions development, *w*_*i*_ is the indicator weight, and *d*_*ij*_ is the standardized value of the three-level indicators. The secondary indicators are composed of tertiary indicators, and the scores of the secondary indicators are calculated according to formula (2). Similarly, the comprehensive score of the primary indicators is calculated based on the scores of the secondary indicators, reflecting the difference in the comprehensive scores of urban and rural elderly-care institutions.

#### Analysis of urban-rural differences

##### Time series data processing

Using the data from the initial year, the second year, the third year, and the data from the final year of the study as the numerator, and the data from the initial year as the denominator, the changes in the scores of the various indicators for urban and rural elderly care institutions were measured over the years. The advantage of this method of processing is that it facilitates comparison and does not require standardization of the data. Some scholars have used this method for data analysis, which can better reflect the trend of data changes (54). Of course, this method also has the disadvantage that if the data for the initial year is missing for individual cities, the data for the second year will need to be used as the initial year data.


(3) $$T_{i}=\{\begin{array}{l} S_{n-1}/S_{n-1},i=1\\ S_{n}/S_{n-1},i=2,\ldots ,n \end{array}$$

Where, *Ti* is the time-series data processing value, *S*_*n*_ is the year of data following the initial value, and Sn-1 is the initial value.

##### Proportional data processing

Proportional data is a measure of the structure of the indicators for elderly care institutions. As the data is in the form of percentages, the proportion data for elderly care institutions in urban areas can be directly subtracted from the proportion data for rural areas to give the difference between the two.


(4) $$\begin{array}{l} P_{i}=P_{u}-P_{r} \end{array}$$

Where, *P*_*i*_ is the difference in proportional data, *P*_*u*_ is the proportional data of elderly care institutions in urban areas and *P*_*r*_ is the proportional data of elderly care institutions in rural areas.

##### Composite score data processing

As the composite score data is dimensionless and the indicators can be compared with each other, the two will be scaled here to reflect the difference between the urban and rural composite scores.


(5) $$\begin{array}{l} Z_{j}=Z_{u}/Z_{r} \end{array}$$

Where, *Z*_*j*_ is the ratio of the composite scores of urban and rural elderly-care institutions, *Z*_*u*_ is the composite score of elderly-care institutions in urban areas, and *Z*_*r*_ is the composite score of elderly-care institutions in rural areas.

#### Spatial autocorrelation analysis

##### Global spatial autocorrelation

Spatial autocorrelation is the degree to which the value of a geographical phenomenon or attribute on a regional unit is correlated with the value of the same phenomenon or attribute in a neighboring geographical unit, and is divided into global spatial autocorrelation and local spatial autocorrelation. The global spatial autocorrelation measure is mainly measured by Moran's index (Moran'I), which is calculated by the following formula (55).


(6) $$\begin{array}{l} I=\left(\frac{n}{\underset{i}{\sum }\underset{j}{\sum }W_{ij}}\right)\left(\frac{\underset{i}{\sum }\underset{j}{\sum }W_{ij}\left(x_{i}-\bar{x}\right)\left(y_{i}-ȳ\right)}{\underset{i}{\sum }{\left(x_{i}-\bar{x}\right)}^{2}}\right) \end{array}$$

Where, *W*_*ij*_ is the spatial matrix; n is the number of cells in the region and xi is the observation of the *i*^*th*^ cell; is the mean value of the observation. The expected value of Moran's index (Moran'I) is


(7) $$\begin{array}{l} E\left(I\right)=-1/\left(n-1\right) \end{array}$$

A positive Moran's Index (Moran'I), provided that it passes the significance test, indicates a spatially significant clustering of regions with high combined scores of elderly care institutions; a positive Moran'I indicates a significant spatial difference between the combined scores of elderly care institutions in a region and its surrounding areas.

##### Local spatial autocorrelation

Local spatial autocorrelation is used to reveal the heterogeneous characteristics of geospatial differences in order to fully reflect the changing trends of regional differences between urban and rural elderly institutions, and is usually measured using Local Moran'I. Local Moran'I is a decomposed form of the global Moran index, which is used to measure the degree of spatial difference and the significance of the difference between the value of an attribute of a spatial unit and its It is used to measure the degree of spatial variation and the significance of the variation between an attribute value of a spatial unit and its surrounding area. For a spatial unit *i*, its Local Moran'I is defined as.


(8) $$\begin{array}{l} I_{i}=z_{i}\underset{i}{\sum }W_{ij}z_{j} \end{array}$$

Where, *z*_*i*_ and *z*_*j*_ are the normalized values of the observations on region *i* and region *j*; W_*ij*_ is the spatial weighting. The local spatial autocorrelation reflects the aggregated score clustering characteristics of elderly institutions through the LISA clustering map.

## Analysis of the results

### Spatial pattern of development of urban and rural elderly care institutions

#### Composite score for urban and rural elderly care institutions

**Figure 3:**
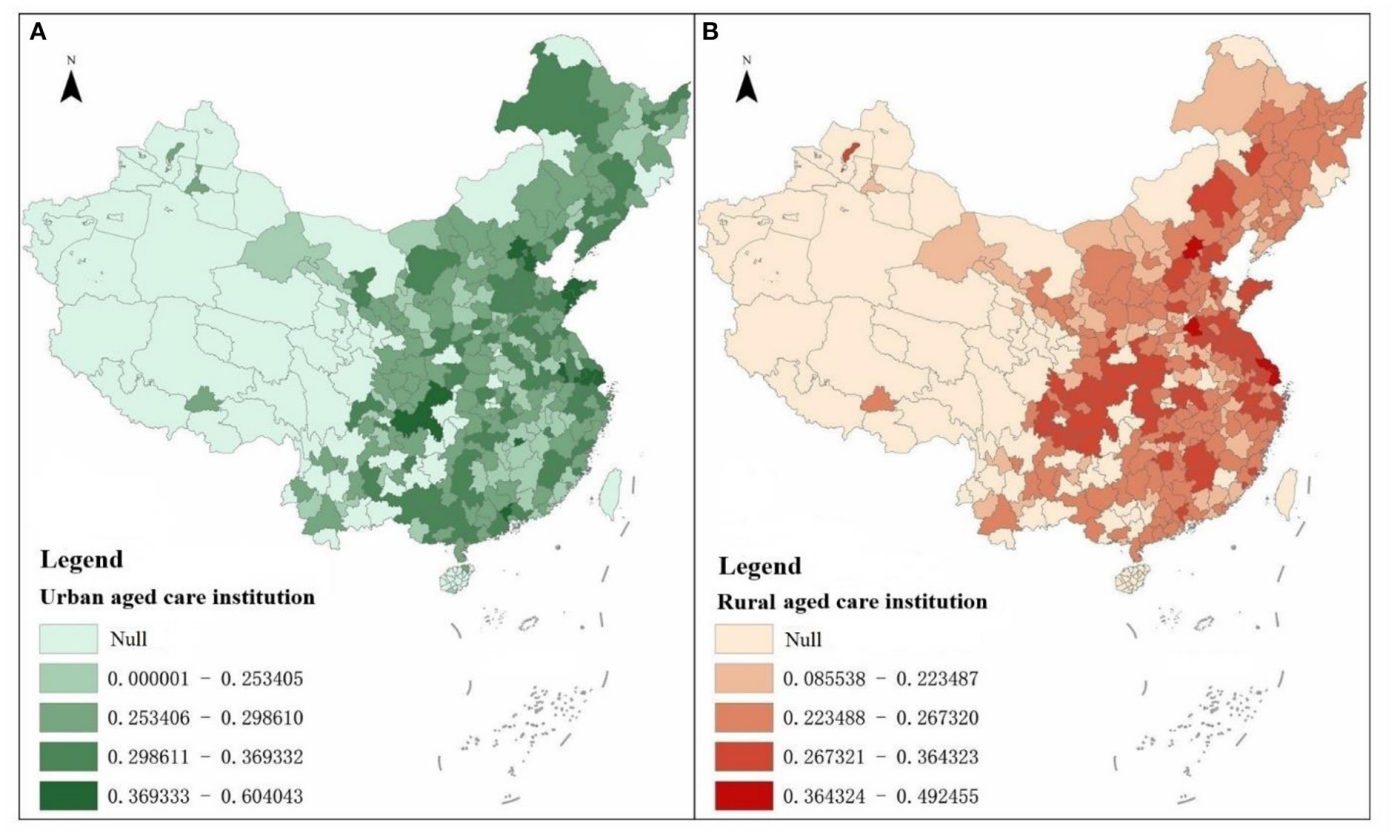
**(A, B)** Schematic of the overall scores of urban and rural elderly care institutions.

Using the composite score measure, the composite score for the development of 276 urban and rural elderly-care institutions in 2016 was measured to reveal the spatial pattern of the development of urban and rural elderly-care institutions (Figure 3, Table 2).

**Table 2:** Ranking of the overall scores of urban and rural elderly care institutions in the four regions (%).

**Ranking**	**Northeast China**		**Eastern region**		**Central region**		**Western region**	
	**Urban**	**Rural**	**Urban**	**Rural**	**Urban**	**Rural**	**Urban**	**Rural**
Top 20%	2.54	0.00	9.42	10.14	3.99	5.07	3.99	4.71
Middle 60%	7.25	10.51	18.48	17.75	15.22	15.22	19.20	16.67
Bottom 20%	2.17	1.45	3.26	3.26	8.70	7.61	5.80	7.61

The top 10 cities with the highest overall scores in terms of city-region elderly-care institutions were Shanghai, Tianjin, Chongqing, Beijing, Nanjing, Suzhou, Guangzhou, Qingdao, Yantai and Xinyu in that order. Among the top 20% of cities in terms of overall score 7, 26, 11 and 11 are in the northeast, east, central and west regions respectively, accounting for 2.54, 9.42, 3.99, and 3.99% respectively. From the cities ranked between 21 and 60% of the overall score, the western region accounted for 53 cities, accounting for 19.20%, and the eastern region followed closely behind, accounting for 51 cities accounting for 18.48%. The central region accounts for 24 cities, or 8.70%, of the bottom 20% of cities in the ranking. Overall, the eastern region has seen rapid development of urban elderly-care institutions, while the northeastern region has lagged behind in relative terms, with the central and western regions in the middle of the pack.

In terms of the overall scores of elderly institutions in rural areas, the top 10 cities with the highest overall scores were Beijing, Shanghai, Heze, Nantong, Chongqing, Xuzhou, Ganzhou, Nanchong, Jining and Taizhou in that order. Among the top 20% of cities with composite scores, 0, 28, 14, and 13 are in the northeast, east, central and west regions respectively, accounting for 0.00, 10.14, 5.07, and 4.71% respectively. For cities with a combined score ranking between 21 and 60%, there were 29, 49, 42 and 46 in the northeast, east, central and west regions respectively. Among the cities with a combined score in the bottom 20%, there were 4, 9, 21, and 21 in the northeast, east, central and west regions respectively. Overall, the eastern region has the highest score for rural elderly institutions, while the other three regions have relatively low scores.

In terms of the differences in the overall scores of urban and rural elderly-care institutions, firstly, the overall scores of elderly-care institutions in urban areas have a clear pointing to large-cities and mega-cities, and among the top 20 elderly-care institutions in terms of overall scores, municipalities directly under the central government, provincial capitals, sub-provincial cities and large cities with developed economies on the eastern coast account for most of them, while the overall scores of elderly-care institutions in rural areas do not have a clear pointing to large cities. Secondly, cities with higher overall scores for elderly care institutions in urban areas or rural areas have a clear eastern coastal pointing, and the level of overall scores in the eastern regions is generally higher than that in the northeastern and central and western regions. Thirdly, the variation in the development of institutions in rural areas is much higher than that in urban areas. 276 cities have a coefficient of variation of 0.2813 for the overall score of institutions in rural areas, which is much higher than that of 0.1634 in urban areas.

#### Comparison of composite scores for secondary indicators

Looking at the 2010 and 2016 scores for the secondary indicator of elderly care, urban elderly care institutions are growing significantly faster than rural ones. In terms of hardware facilities, urban aged-care institutions are developing rapidly, while rural areas are on the contrary showing a shrinking development trend. In terms of software environment, the caregivers in urban aged-care institutions already scored higher than those in rural areas in 2010, and the gap between urban and rural areas widened further in 2016. In terms of clients served by elderly care institutions, urban areas still score higher than rural areas (Table 3).

**Table 3:** Secondary indicator scores for urban and rural aged care institutions.

**Year**	**Construction of facilities**		**Nursing staff of elderly-care institutions**		**Service users of elderly-care institutions**	
**2010**	**Urban**	**Rural**	**Urban**	**Rural**	**Urban**	**Rural**
	2.29	3.80	26.34	25.83	42.32	36.30
2016	Urban	Rural	Urban	Rural	Urban	Rural
	3.11	3.35	30.38	19.76	45.80	39.86

Firstly, the scores of the secondary indicators for the construction of elderly care institutions. In terms of spatial distribution, the scores of the secondary indicator of the construction of elderly care institutions' facilities in the eastern urban areas are higher than those in the central and western regions, while the differences in the scores of the secondary indicator of the construction of elderly care institutions' facilities in the rural areas in the east, central and western regions are relatively smaller. Among the top 20% of cities, 4.71, 10.87, 1.45, and 2.90% of urban areas in the northeast, east, central and west regions respectively, and 0.00, 7.61, 6.88, and 5.43% of rural areas respectively. Among the cities scoring in the middle 60%, the urban areas in the Northeast, East, Central and West regions accounted for 7.25, 17.75, 19.57, and 15.58% respectively, while the rural areas accounted for 9.78, 19.20, 17.75, and 13.41% respectively. Among the bottom 20% of cities in the four regions of Northeast, East, Central and West, urban areas accounted for 0.00, 2.54, 6.88, and 10.51% respectively, while rural areas accounted for 2.17, 4.35, 3.26, and 10.14% respectively.

Secondly, the scores of the secondary indicator for caregivers in elderly institutions. In terms of spatial distribution, the regions with higher scores on the secondary indicator of nursing staff in elderly institutions are mainly concentrated in urban areas in the east; the difference in scores on the secondary indicator of nursing staff in elderly institutions between rural areas in the east and rural areas in the west is relatively small. Among the top 20% of cities, 0.72, 7.97, 4.35, and 6.88% of urban areas in the northeast, east, central and west regions respectively, and 1.45, 7.25, 3.99, and 7.25% of rural areas respectively. Among the cities scoring in the middle 60%, the urban areas in the Northeast, East, Central and West regions accounted for 8.70, 18.12, 16.67, and 16.67% respectively, while the rural areas accounted for 9.42, 17.03, 17.39, and 16.30% respectively. Among the bottom 20% of cities in the four regions of Northeast, East, Central and West, urban areas accounted for 2.54, 5.07, 6.88, and 5.43% respectively, while rural areas accounted for 1.09, 6.88, 6.52, and 5.43% respectively.

Thirdly, the scores of the secondary indicators of service recipients of elderly care institutions. In terms of spatial distribution, the regions with higher scores on the secondary indicator of service recipients of elderly-care institutions are mainly concentrated in the rural areas in the east; the scores on the secondary indicator of service recipients of elderly-care institutions are higher in the urban areas in the east than in the central and western regions, and the central and western regions are higher than the eastern regions. Among the top 20% of cities, 1.81, 7.61, 4.71, and 5.80 of urban areas in the northeast, east, central and west regions respectively, and 0.36, 13.04, 3.26, and 3.26% of rural areas respectively. Among the cities scoring in the middle 60%, the urban areas in the northeast, east, central and west regions accounted for 8.33, 20.29, 15.94, and 15.58% respectively, while the rural areas accounted for 10.14, 14.86, 18.84, and 16.30% respectively. Among the bottom 20% of cities in the four regions of Northeast, East, Central and West, urban areas accounted for 1.81, 3.26, 7.25, and 7.61% respectively, while rural areas accounted for 1.45, 3.26, 5.80, and 9.42% respectively.

### Time series changes in the development of urban and rural elderly care institutions

#### Time series variation in the development of urban elderly care institutions

Urban area elderly care institutions in 276 cities in China have developed rapidly in terms of both hardware facilities and software conditions (Table 4). In terms of hardware facilities, from 2010 to 2016, the number of elderly institutions in urban areas increased from 5,510 to 8,474, the number of beds at the end of the year increased from 576,700 to 1,310,800, and the floor area of elderly institutions also increased from 10,735,800 square meters to 26,866,700 square meters. In terms of software conditions, from 2010 to 2016, the number of year-end employees in aged-care institutions increased from 75,000 to 144,600, and the proportion of university-educated nursing staff in aged-care institutions rose from 16.54 to 24.99%. In terms of admission to elderly care institutions, the number of people in elderly care institutions at the end of the year rose from 364,900 to 650,600 between 2010 and 2016. In terms of the structure of people staying in elderly care institutions, the proportion of self-financed people rose rapidly, while the proportion of “three no-good” people fell rapidly. In terms of self-care, the proportion of people who are able to take care of themselves has decreased, from 62.24 to 57.80% between 2010 and 2016, while the proportion of people who are semi-self-care and completely unable to take care of themselves has increased significantly, with a cumulative increase of 17.48 percentage points for both.

**Table 4:** Time series changes in evaluation indicators of elderly care institutions in urban areas (unit: units, 10,000 square meters, 10,000 beds, persons, days, %).

**Urban**	**2010**	**2011**	**2012**	**2013**	**2014**	**2015**	**2016**
Number of elderly care institutions	5,510	5,645	6,277	6,915	7,452	7,474	8,474
GFA of elderly care facilities	1,074	1,321	1,533	1,864	2,269	2,459	2,869
Number of beds at end of year	57.67	63.60	74.16	99.23	106.45	116.84	131.08
Number of employees at end of year	7.50	8.07	9.18	10.55	11.92	12.80	14.46
Proportion of female caregivers	58.67	56.79	57.22	57.63	57.56	58.85	59.66
Percentage of university education	16.54	15.09	16.26	20.48	22.80	24.69	24.99
Proportion of persons aged 35 and under	25.53	25.32	26.37	26.75	26.59	26.39	25.65
Proportion of persons aged 36–55 years	51.69	48.78	53.00	56.70	58.51	60.75	64.24
Proportion of persons aged 56 and over	22.78	25.90	20.63	16.75	15.81	13.38	10.11
Average number of days in elderly care institution per year	237	247	248	243	239	239	236
Number of people in elderly care institution at the end of the year	36.49	38.73	42.83	96.77	56.80	58.59	65.06
Proportion of beneficiaries	2.61	3.15	2.64	2.70	2.22	1.78	1.91
Proportion of “three noughts”	31.97	31.55	30.55	21.98	24.44	20.84	19.67
Proportion of self-financing staff	65.07	65.30	66.45	73.87	73.34	77.39	78.43
Proportion of fully self-care workers	62.24	56.93	61.27	62.66	58.08	57.13	57.80
Proportion of semi-self-care persons	13.55	18.23	18.03	19.88	21.00	22.66	24.53
Proportion of people who cannot care for themselves	8.27	9.26	10.55	12.03	11.14	13.68	14.77

#### Time series variation in the development of rural elderly care institutions

Elderly care institutions in rural areas in 276 cities in China have seen some development in terms of hardware and software conditions, but overall, there is a sharp downward trend (Table 5). In terms of hardware, from 2010 to 2016, the number of elderly institutions and the number of beds at the end of the year in rural areas decreased from 28,520 and 2,092,100 to 14,773 and 1,680,600 respectively, and the floor area of elderly institutions also decreased from 31,551,400 square meters to 31,347,800 square meters. In terms of software conditions, from 2010 to 2016, the number of year-end employees in elderly institutions decreased from 131,900 to 106,400, and the proportion of university-educated nursing staff in elderly institutions increased from 9.08 to 16.79%. In terms of admission to elderly care institutions, the number of people in elderly care institutions at the end of the year fell sharply from 1,691,200 to 1,037,300 between 2010 and 2016. In terms of the structure of people staying in elderly care institutions, the proportion of self-financed people rose rapidly, while the 2016, proportion of “three no-good” people increased slightly. In terms of self-care, the proportion of people who are able to take care of themselves has decreased, from 76.29 to 69.55% between 2010 and while the proportion of people who are semi-self-care and completely unable to take care of themselves has increased significantly, with a cumulative increase of 7.83 percentage points for both.

**Table 5:** Time series changes in evaluation indicators of elderly care institutions in Rural areas (unit: units, 10,000 square meters, 10,000 beds, persons, days, %).

**Rural**	**2010**	**2011**	**2012**	**2013**	**2014**	**2015**	**2016**
Number of elderly care institutions	28,520	29,100	29,736	22,523	19,144	15,081	14,773
GFA of elderly care facilities	3,155	4,384	4,798	3,876	3,366	2,963	3,135
Number of beds at end of year	209.21	226.11	244.68	206.39	209.72	167.69	168.06
Number of employees at end of year	13.19	14.29	14.89	14.32	12.42	10.46	10.64
Proportion of female caregivers	44.37	45.16	45.48	48.71	55.95	63.33	55.80
Percentage of university education	9.08	11.46	10.54	15.85	14.13	15.61	16.79
Proportion of persons aged 35 and under	20.07	20.90	20.71	25.15	24.58	23.55	21.49
Proportion of persons aged 36–55 years	63.07	62.11	63.18	68.30	65.55	65.45	66.10
Proportion of persons aged 56 and over	7.44	7.21	5.60	5.83	6.61	6.28	7.34
Average number of days in elderly care institution per year	226	255	267	261	253	249	246
Number of people in elderly care institution at the end of the year	169.12	179.12	186.33	137.47	144.57	106.71	103.73
Proportion of beneficiaries	3.37	2.96	2.99	3.17	3.62	3.47	3.54
Proportion of “three noughts”	67.47	63.18	57.41	72.20	75.74	72.94	72.14
Proportion of self-financing staff	5.62	5.24	7.36	22.09	14.49	16.71	18.88
Proportion of fully self-care workers	76.29	74.11	70.32	72.20	71.74	70.27	69.55
Proportion of semi-self-care persons	13.38	14.83	14.64	18.62	17.14	18.30	19.24
Proportion of people who cannot care for themselves	4.17	4.17	4.53	6.65	4.96	5.27	6.14

#### Temporal changes in the differences of urban and rural elderly care institutions

**Figure 4:**
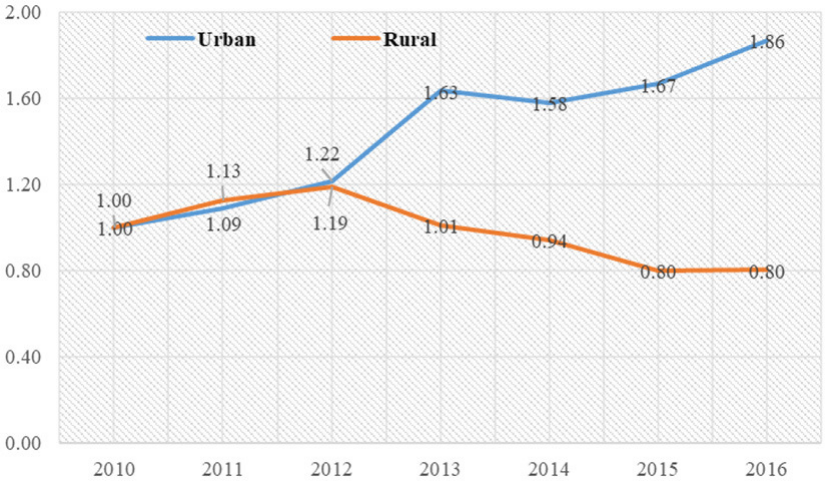
Temporal change in the development of elderly care institutions in urban and rural areas.

Comparing the time-series variation in differences in the development of elderly-care institutions in urban areas and rural areas across 276 cities, it was found that:

First, in terms of quantitative indicators. During the study period, the indicators of the number of elderly-care institutions in urban areas strengthened across the 276 cities in China, while the indicators of the number of elderly-care institutions in rural areas contracted across the board (Table 6). Taking the first year of the study period as the base period, comparing the differences in the changes of elderly-care institutions in urban and rural areas, the number of elderly-care institutions, the number of employees at the end of the year, and the number of people in elderly-care institutions at the end of the year, in addition to the indicator of the average number of days in elderly-care institutions per year, showed that elderly-care institutions in urban areas were developing rapidly, while those in rural areas were in an overall trend of contraction. Taking the mean values of the six quantitative indicators, a more pronounced time-series trend of urban-rural differences was found (Figure 4). 2010–2012 saw a small difference in the growth of the mean value of the quantitative indicators between urban and rural areas, but from 2013–2016 the difference in the growth of the mean value of the quantitative indicators between the two tended to widen. Instead of increasing, the number of institutions in rural areas shrank by nearly 20 percentage points relative to the study base period, while the number of institutions in urban areas expanded by 1.86 times, making the difference between urban and rural areas more significant in the study end period.

**Table 6:** Time-series variation in the number of indicators for the number of elderly care institutions in urban and rural areas.

**Region**	**Quantitative indicators**	**2010**	**2011**	**2012**	**2013**	**2014**	**2015**	**2016**
Urban	Number of elderly care institutions	1.00	1.02	1.14	1.25	1.35	1.36	1.54
	GFA of elderly care facilities	1.00	1.23	1.43	1.74	2.11	2.29	2.67
	Number of beds at end of year	1.00	1.10	1.29	1.72	1.85	2.03	2.27
	Number of employees at end of year	1.00	1.08	1.22	1.41	1.59	1.71	1.93
	Average number of days in elderly care institution per year	1.00	1.04	1.04	1.02	1.01	1.00	1.00
	Number of people in elderly care institution at the end of the year	1.00	1.06	1.17	2.65	1.56	1.61	1.78
Rural	Number of elderly care institutions	1.00	1.02	1.04	0.79	0.67	0.53	0.52
	GFA of elderly care facilities	1.00	1.39	1.52	1.23	1.07	0.94	0.99
	Number of beds at end of year	1.00	1.08	1.17	0.99	1.00	0.80	0.80
	Number of employees at end of year	1.00	1.08	1.13	1.09	0.94	0.79	0.81
	Average number of days in elderly care institution per year	1.00	1.13	1.18	1.15	1.12	1.10	1.09
	Number of people in elderly care institution at the end of the year	1.00	1.06	1.10	0.81	0.85	0.63	0.61

Second, in terms of proportional indicators. In terms of proportional indicators, there are significant differences between some of the proportional indicators of urban and rural elderly care institutions (Table 7). Firstly, in terms of the gender structure of workers in elderly care institutions, the proportion of female caregivers in both urban and rural areas ranged from 56 to 60%. Secondly, in terms of the proportion of workers with higher education, the proportion of workers with higher education in both urban and rural elderly-care institutions is not high, being <25%; the proportion of workers with higher education in both urban and rural elderly-care institutions is increasing, but the proportion of workers with higher education working in rural elderly-care institutions is still lower than that in urban areas. Thirdly, the age structure of workers in urban and rural elderly-care institutions tends to be similar, with the proportion of young people (elderly 35 and below), middle-elderly people (elderly 36–55) and middle-elderly and elderly people (elderly 56 and above) ranging from 20–26%, 64–66, and 7–10%, with middle-elderly people constituting the main force of workers in elderly-care institutions. Fourthly, there is a significant difference in the structure of people living in residential care institutions between urban and rural areas in terms of the nature of the people living there. The proportion of “three no-good” people in old-age institutions in rural areas is absolutely dominant, while the proportion of self-funded people in urban areas is absolutely dominant; the proportion of beneficiaries in old-age institutions in rural areas is slightly higher than that in urban areas. Fifthly, in terms of the ability of people to take care of themselves, the proportion of people who take care of themselves completely tends to decline in both urban and rural areas, while the proportion of people who take care of themselves partially and cannot take care of themselves is rapidly increasing. The proportion of semi-self-care and non-self-care residents in urban institutions is higher than that in rural areas, while the proportion of fully self-care residents is lower than that in rural areas.

**Table 7:** Temporal variation in indicators of differences in the proportion of elderly institutions in urban and rural areas (unit: %).

**Proportional indicators**	**2010**	**2011**	**2012**	**2013**	**2014**	**2015**	**2016**
Proportion of female caregivers	14.30	11.63	11.74	8.91	1.62	−4.48	3.86
Percentage of university education	7.46	3.63	5.72	4.62	8.67	9.09	8.20
Proportion of persons elderly 35 and under	5.46	4.42	5.66	1.60	2.01	2.83	4.16
Proportion of people elderly 36–55 years	−11.38	−13.33	−10.18	−11.60	−7.04	−4.70	−1.85
Proportion of persons elderly 56 and over	15.34	18.69	15.03	10.93	9.20	7.10	2.77
Proportion of beneficiaries	−0.76	0.19	−0.35	−0.47	−1.40	−1.70	−1.64
Proportion of “three noughts”	−35.50	−31.63	−26.86	−50.22	−51.30	−52.10	−52.47
Proportion of self–financing staff	59.45	60.06	59.09	51.77	58.86	60.68	59.55
Proportion of fully self–care workers	−14.05	−17.18	−9.05	−9.54	−13.66	−13.14	−11.75
Proportion of semi–self–care persons	0.16	3.40	3.39	1.26	3.86	4.36	5.29
Proportion of people who cannot care for themselves	4.10	5.08	6.02	5.38	6.18	8.41	8.63

Third, reason analysis. Firstly, the ability to build and maintain hardware facilities in rural areas has declined significantly over the study period, indicating a greater pressure on the continued development of rural institutions and their maintenance. Secondly, there is a regressive trend in software conditions in rural areas. During the study period, the number of workers employed in rural institutions fell steeply, and the number of older people staying in rural institutions fell rapidly, in contrast to urban areas, which showed rapid growth. Thirdly, the proportion of “three no-good” people living in institutions in rural areas has not decreased, but has increased rapidly, which has put a lot of pressure on local finances and social care. Fourthly, with the combined impact of an aging population, advanced aging, empty nesting families and the weakening of traditional elderly care functions, the problem of elderly care services for the empty nesters, the elderly alone and the elderly disabled is becoming more and more acute in both urban and rural areas. However, due to factors such as the weak financial ability of the elderly, this has led to a low proportion of self-funded persons staying in elderly care institutions in rural areas and a decrease in occupancy. Fifthly, the professional quality of the staff working in elderly care institutions is not high in both urban and rural areas, and this is not compatible with the professional elderly care services. Sixthly, elderly care institutions are not the first choice for young people, resulting in a lack of motivation among service providers. In both urban and rural areas, the proportion of young people working in elderly care institutions is low and remains below 25%, while the proportion of middle-elderly workers elderly 36–55 is increasing, meaning that the age structure of workers tends to be middle-elderly.

### Spatial autocorrelation analysis of urban and rural elderly care institutions

#### Analysis of global spatial autocorrelation

A global spatial autocorrelation analysis was conducted on the combined scores of elderly institutions development in urban and rural areas in 2010 and 2016 (Table 8). The results show that the Moran's I index for the combined scores of elderly institutions in both urban and rural areas is positive, indicating that the combined scores of elderly institutions are spatially correlated or clustered, i.e., the combined scores of elderly institutions in an area are related to the location of that area. As the Moran's I value varies between −1 and 1, the higher the absolute value of Moran's I, the stronger the spatial correlation. z scores range from 24.00 to 26.00, indicating that the distribution of the combined scores of elderly institutions exhibits a clustering pattern.

**Table 8:** Global spatial autocorrelation analysis.

**Index**	**Urban (2010)**	**Rural (2010)**	**Urban (2016)**	**Rural (2016)**
Moran'I	0.3725	0.3662	0.3464	0.3677
z–Score	25.8854	25.4472	24.0826	25.5451
P–Value	0.0000	0.0000	0.0000	0.0000

In terms of changes in the Moran'I index, the index for urban areas decreased from 0.3725 to 0.3464 between 2010 and 2016, indicating a slight weakening of the spatial agglomeration of elderly-care institutions in urban areas, while the Moran'I index in rural areas increased from 0.3662 to 0.3677, indicating a slight increase in the spatial agglomeration of elderly-care institutions in rural areas. In terms of the Moran 'I index for the combined scores of urban and rural elderly institutions, the spatial agglomeration of elderly institutions in urban areas was slightly higher than that in rural areas in 2010; in 2016, the spatial agglomeration of elderly institutions in rural areas overtook that in urban areas.

In the significance level test, the global spatial autocorrelation of the combined scores of urban and rural elderly care institutions in 2010 and 2016 both passed the test with a *p*-value of 0.0000, which has a high significance level. This indicates that the analysis of the global spatial autocorrelation of the combined scores of elderly care institutions is not randomly generated and the results are highly reliable.

#### Analysis of local spatial autocorrelation

**Figure 5:**
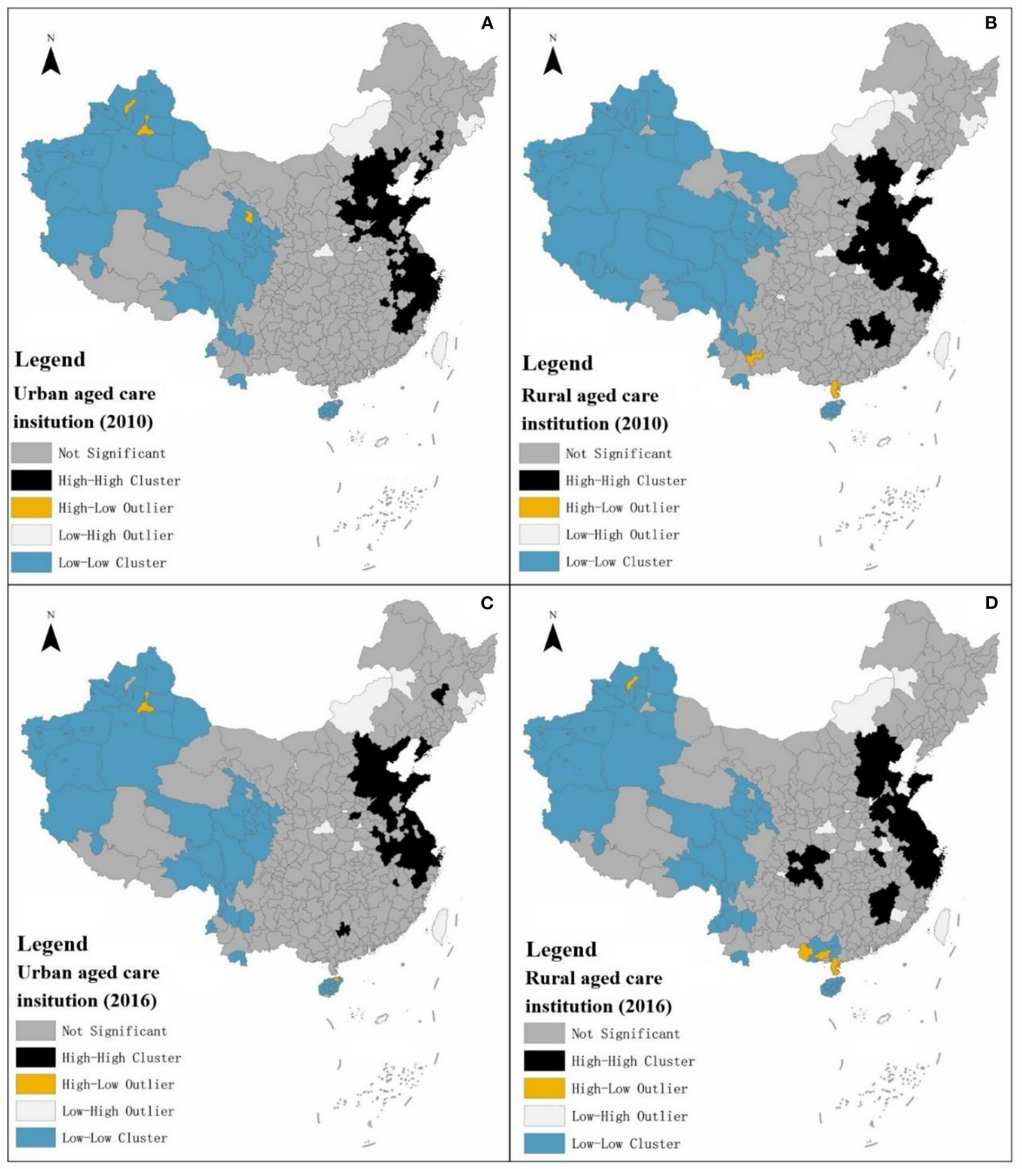
**(A–D)** Schematic diagram of the local spatial autocorrelation analysis of the development of urban and rural elderly institutions.

Local spatial autocorrelation analysis was used to reveal the spatial and temporal patterns of the development of elderly institutions in urban and rural areas (Figure 5).

The local spatial autocorrelation classifies the combined scores of urban or rural elderly-care institutions in each city as insignificant, High-High Cluster, High-Low Outlier, Low-High Outlier and Low-Low Cluster. Due to the lack of data, cities with missing data are assigned a value of zero in the process of local spatial autocorrelation analysis, especially for contiguous areas, resulting in the geospatial phenomenon of contiguous low-low clusters. Due to the lack of actual data support, this type of low-low agglomeration analysis is of little value. High-Low Outlier or Low-High Outlier tend to occur in contiguous areas of low-low agglomeration or in areas with insignificant contiguity, and are less numerous compared to High-High or Low-Low agglomeration. Therefore, from the above, the analysis will focus on the local spatial autocorrelation of high-high clustering in urban and rural areas for elderly care institutions.

First, local spatial autocorrelation analysis of elderly institutions in urban areas. Seventy one cities with high-high concentration type of elderly institutions in urban areas were analyzed in 2010, with 51, 15, 0, and 5 in the eastern, central, western and northeastern regions respectively.

In 2016, the number of cities with high-high concentration type of elderly institutions decreased to 64, of which, 48, 12, 1, and 3 in the eastern, central, western region and northeastern region accounted for 48, 12, 1, and 3 respectively. From the perspective of city cluster, the cities with high-high concentration type of local spatial autocorrelation analysis of elderly institutions in urban areas from 2010 to 2016 were mainly concentrated in the Beijing-Tianjin-Hebei city cluster, Shandong Peninsula city cluster and Yangtze River Delta city cluster, and showed relatively stable characteristics (Table 9).

**Table 9:** Distribution of high–high agglomeration types for local spatial autocorrelation analysis in urban.

**Region**	**Urban (2010)**	**Urban (2016)**
Eastern region	Beijing, Tianjin, Shijiazhuang, Tangshan, Handan, Xingtai, Baoding, Zhangjiakou, Cangzhou, Langfang, Chengde, Shanghai, Nanjing, Wuxi, Xuzhou, Changzhou, Suzhou, Taizhou, Lianyungang, Huaian, Yangzhou, Zhenjiang, Hangzhou, Ningbo, Jiaxing, Huzhou, Wenzhou, Taizhou, Shaoxing, Jinhua, Zhoushan, Lishui, Jinan, Qingdao, Zibo, Zaozhuang, Dongying, Yantai, Weifang, Jining, Taian, Weihai, Rizhao, Laiwu, Dezhou, Liaocheng, Binzhou, Heze, Sanming, Nanping	Beijing, Tianjin, Shijiazhuang, Tangshan, Handan, Xingtai, Baoding, Zhangjiakou, Cangzhou, Langfang, Shanghai, Nanjing, Wuxi, Xuzhou, Changzhou, Suzhou, Nantong, Yancheng, Lianyungang, Huaian, Yangzhou, Zhenjiang, Hangzhou, Ningbo, Jiaxing, Huzhou, Shaoxing, Jinhua, Zhoushan, Lishui, Jinan, Qingdao, Zibo, Zaozhuang, Dongying, Yantai, Weifang, Jining, Taian, Weihai, Rizhao, Laiwu, Dezhou, Liaocheng, Binzhou, Heze, Qinhuangdao, Hengshui
Central region	Hefei, Bengbu, Maanshan, Tongling, Huangshan, Xuancheng, Yingtan, Chizhou, Taiyuan, Changzhi, Jinzhong, Nanchang, Jingdezhen, Zhengzhou, Xinxiang	Hefei, Bengbu, Maanshan, Tongling, Huangshan, Xuancheng, Wuhu, Anqing, Fuyang, Suizhou, Liu'an, Yingtan
Western region	–	Hezhou
Northeast China	Dalian, Anshan, Fushun, Chaoyang, Liaoyuan	Changchun, Yingkou, Dalian

Second, local spatial autocorrelation analysis of elderly institutions in rural areas. in 2010, there were 80 cities with high-high concentration type of elderly institutions in rural areas, with the eastern region, central region, western region and northeastern region accounting for 50, 29, 0 and 1 respectively; in 2016, the number of cities with high-high concentration type decreased to 66, of which the eastern region, central region, western region and northeastern region accounted for 52, 11, 3, and 0 respectively. From the perspective of city clusters, cities with high-high agglomeration type of local spatial autocorrelation analysis of elderly institutions in rural areas from 2010 to 2016 were mainly concentrated in the Beijing-Tianjin-Hebei city cluster, Shandong Peninsula city cluster and Yangtze River Delta city cluster (Table 10).

**Table 10:** Distribution of high–high agglomeration types for local spatial autocorrelation analysis in rural.

**Region**	**Rural (2010)**	**Rural (2016)**
Eastern region	Beijing, Tianjin, Tangshan, Qinhuangdao, Handan, Xingtai, Baoding, Zhangjiakou, Chengde, Cangzhou, Langfang, Hengshui, Shanghai, Nanjing, Wuxi, Xuzhou, Changzhou, Nantong, Lianyungang, Huaian, Yancheng, Yangzhou, Zhenjiang, Taizhou, Suqian, Hangzhou, Ningbo, Wenzhou, Jiaxing, Huzhou, Shaoxing, Jinhua, Zhoushan, Taizhou, Lishui, Jinan, Qingdao, Zibo, Zaozhuang, Yantai, Weifang, Jining, Tai'an, Weihai, Laiwu, Linyi, Dezhou, Liaocheng, Binzhou, Heze	Beijing, Tianjin, Shijiazhuang, Tangshan, Qinhuangdao, Handan, Xingtai, Baoding, Zhangjiakou, Chengde, Cangzhou, Langfang, Hengshui, Shanghai, Nanjing, Wuxi, Xuzhou, Changzhou, Suzhou, Nantong, Lianyungang, Huaian, Yancheng, Yangzhou, Zhenjiang, Taizhou, Suqian, Hangzhou, Ningbo, Wenzhou, Jiaxing, Huzhou, Shaoxing, Jinhua, Quzhou, Zhoushan, Taizhou, Lishui, Qingdao, Zibo, Dongying, Yantai, Jining, Taian, Weihai, Rizhao, Laiwu, Linyi, Dezhou, Liaocheng, Binzhou, Heze
Central region	Taiyuan, Hefei, Wuhu, Bengbu, Huainan, Maanshan, Tongling, Anqing, Huangshan, Chuzhou, Fuyang, Suizhou, Liuan, Xuancheng, Ganzhou, Ji'an, Zhengzhou, Kaifeng, Pingdingshan, Xinxiang, Puyang, Xuchang, Nanyang, Shangqiu, Xinyang, Zhoukou, Zhumadian, Hengyang, Chenzhou	Wuhu, Bengbu, Chuzhou, Fuyang, Suizhou, Xuancheng, Ganzhou, Ji'an, Fuzhou, Shangqiu, Huanggang
Western region	–	Chongqing, Nanchong, Dazhou
Northeast China	Dalian	–

Third, there are differences in local spatial autocorrelation between urban and rural elderly-care institutions. Quantitatively, the number of local spatial autocorrelation high-high clusters of elderly-care institutions in rural areas is slightly higher than that in urban areas, with nine and two more cities in 2010 and 2016 respectively. In terms of spatial distribution, the local spatial autocorrelation of high-high concentration of elderly institutions in both urban and rural areas points to the Beijing-Tianjin-Hebei city cluster, Shandong Peninsula city cluster and Yangtze River Delta city cluster, but the spatial scope of local spatial autocorrelation of high-high concentration of elderly institutions in rural areas is slightly wider, with the spatial scope of high-high concentration of elderly institutions in rural areas involving nine provinces and cities, including Beijing, Tianjin, Hebei, Jiangsu, Zhejiang, Shanghai, Anhui, Jiangxi and Chongqing in 2016.

## Discussion and conclusion

### Discussion

Differences in the development of urban and rural institutions are widening, both between and within regions, and most cities are characterized by an evolutionary pattern of “urban progress and rural regression,” implying that the spatial layout of institutions is becoming more and more urban, with a high-high concentration of institutions in the eastern coastal regions of China. In comparison with Feng and Ma (31), we also find that the “hard power” of urban institutions has improved significantly, while the “soft power” has yet to be improved, although our perspective differs from Feng and Ma (31) paper. Feng and Ma (31) focuses on the characteristics of changes in elderly care institutions in 2015 and 2016 from the perspective of equalization of resources in elderly care services, while we compare the spatial and temporal differences in elderly care institutions in urban and rural China from 2010 to 2016 (31). Wu (2022) and Jiang (2011) analyzed the factors influencing the development of elderly care institutions in China (33, 56). The former reveals that the burden of family care is higher for rural than for urban older people with limitations in their activities of daily living, confirming the evolutionary feature of “urban into rural retreat” derived from our study; the latter reveals the willingness of urban and rural older people to move into elderly care institutions, pointing out that rural older people have very limited income and disposable funds, which severely limits their demand for elderly care institutions. The latter reveals the willingness of older people in both urban and rural areas to move into institutions, pointing out that the limited income and financial resources at their disposal severely constrain their demand for institutions, echoing our findings on the reasons for the decline of rural institutions.

Even in the Yangtze River Delta, the most economically developed region in China, 36.65% of the elderly are willing to pay <RMB 1,000/month for a nursing home, 36.66% are willing to pay RMB 1,000–1999/month and 18.21% are willing to pay RMB 2,000–2,999/month (23). The contradiction between the limited ability to pay and the huge demand for elderly care services for elderly people staying in elderly care institutions is bound to affect the sustainability of the development of public elderly care institutions in urban and rural areas. Due to historical factors, the marketization of elderly institutions in China started late and the marketization of elderly care is not high. In the early stage, China established the five-guarantee household support system to provide food, clothing, housing and medical services for the “three have-nots.” With the increasing aging of the population, China's elderly care institutions began to transform from public institutions of a welfare nature to those with a certain degree of market mechanism, but the overall transformation has been slow, which has resulted in the service protection system of elderly care institutions lagging far behind the actual needs of the elderly. It is imperative to continue to promote the market-oriented reform of elderly care institutions, whether in the form of public-run public, public-private or private-run private institutions. The “9073” model pioneered in Shanghai, China's most developed city for the elderly, where 90% of the elderly are self-cared for by their families and adopt family-based home care, 7% enjoy community-based home care services providing day care and 3% enjoy institutional care, provides a demonstration effect for targeted reform of elderly care institutions. On the one hand, the long-term development of elderly care institutions must introduce market mechanisms to strengthen the endogenous power of elderly care institutions, which is the first driving force for their sustainable development and which ensures the quality of services; on the other hand, the relationship between fairness and efficiency of elderly care services needs to be correctly handled, and the professionalism, income and treatment level of elderly care workers need to be improved, on the premise of making up for the inadequate development of elderly care institutions and the insufficient spatial layout. Thus, the internal quality of elderly care institutions can be continuously improved.

### Conclusions

The article uses comprehensive score measurement, urban-rural difference analysis and spatial autocorrelation analysis to reveal the spatial and temporal evolutionary characteristics of urban-rural differences in the development of urban and rural elderly care institutions, and draws the following main conclusions: First, in terms of spatial pattern, the overall score of elderly care institutions in urban areas shows a “double-high” spatial pattern of higher scores in coastal areas than inland areas, and higher scores in urban areas than in rural areas. In terms of the differences in the scores of secondary indicators, the eastern urban areas have higher scores than the rural areas for the indicators of facilities construction and nursing staff of elderly institutions, while the eastern rural areas have higher scores than their urban counterparts for the indicators of service recipients of elderly institutions. Second, in terms of temporal change, there is a clear “urban progress and rural regression” in the evolution of China's elderly care institutions. Third, in terms of spatial and temporal evolution, there is a clear spatial autocorrelation in the composite scores of urban and rural elderly care institutions in China, and the spatial autocorrelation of the composite scores of elderly care institutions shows a clustering pattern.

## Data availability statement

Publicly available datasets were analyzed in this study. This data can be found here the datasets are publicly available *via* China Civil Affairs Statistical Yearbook Published by China Statistics Press.

## Author contributions

Conceptualization: XL. Methods and writing—original draft preparation: CL. Data collection: YH. Writing—review and editing: XL and CL. All authors contributed to the article and approved the submitted version.
